# SOD1 Gene +35A/C (exon3/intron3) Polymorphism in Type 2 Diabetes Mellitus among South Indian Population

**DOI:** 10.1155/2016/3787268

**Published:** 2016-04-17

**Authors:** K. Nithya, T. Angeline, W. Isabel, A. J. Asirvatham

**Affiliations:** ^1^PG & Research Department of Zoology & Biotechnology, Lady Doak College, Madurai, Tamil Nadu 625 002, India; ^2^Arthur Asirvatham Hospital, Madurai, Tamil Nadu 625 020, India

## Abstract

Superoxide dismutase is an antioxidant enzyme that is involved in defence mechanisms against oxidative stress. Cu/Zn SOD is a variant that is located in exon3/intron3 boundary. The aim of the present study was to investigate whether the Cu/Zn SOD (+35A/C) gene polymorphism is associated with the susceptibility to type 2 diabetes mellitus among south Indian population. The study included patients with type 2 diabetes mellitus (*n* = 100) and healthy controls (*n* = 75). DNA was isolated from the blood and genotyping of Cu/Zn SOD gene polymorphism was done by polymerase chain reaction based restriction fragment length polymorphism method. Occurrence of different genotypes and normal (A) and mutant (C) allele frequencies were determined. The frequency of the three genotypes of the total subjects was as follows: homozygous wild-type A/A (95%), heterozygous genotype A/C (3%), and homozygous mutant C/C (2%). The mutant (C) allele and the mutant genotypes (AC/CC) were found to be completely absent among the patients with type 2 diabetes mellitus. Absence of mutant genotype (CC) shows that the Cu/Zn SOD gene polymorphism may not be associated with the susceptibility to type 2 diabetes mellitus among south Indian population.

## 1. Introduction

Diabetes mellitus is a polygenic metabolic disease characterized by hyperglycemia resulting from defects in both insulin secretion and its action [1]. Both the genetic and the environmental factors are involved in the development of type 2 diabetes mellitus [2]. Oxidative stress and oxidative damage to the tissue are common end points of chronic diseases, such as atherosclerosis, diabetes, and rheumatoid arthritis [3]. It has been demonstrated that oxidative stress in diabetes can be accelerated not only due to increased production of reactive oxygen species (ROS) caused by hyperglycaemia but also by reduced ability of antioxidant defense system caused at least partly by polymorphisms of scavenger enzymes like superoxide dismutase, catalase, and reduced glutathione [4]. Superoxide dismutase (SOD) is considered as a primary enzyme since it is involved in the direct elimination of reactive oxygen species [5]. In mammals, there are 3 isoforms of SOD, copper-zinc SOD (SOD1), manganese SOD (SOD2), and an extracellular form of CuZn-SOD (EC-SOD or SOD3). Each superoxide dismutase is the product of distinct genes but catalyzes the same reaction [6]:

(1) $$\begin{array}{c} 2{O_{2}}^{-}+2H^{+}\overset{SOD}{\to }H_{2}O_{2}+O_{2} \end{array}$$

Among the 3 isoforms, experimental evidences from mice indicate that activity of CuZn-SOD accounts for 50% to 80% of total SOD activity. Mn-SOD accounts for approximately 2% to 12% of total vascular SOD, and EC-SOD accounts for the remainder [7]. SOD1 [EC1.15.1.1] has a molecular mass of about 32,000 Da and has been found in the cytoplasm, nuclear compartments, and lysosomes of mammalian cells [8]. The SOD1 gene has been extensively studied and DNA sequence changes in the gene can be detected in about 0.3% of humans worldwide [9]. It has been hypothesized that mutations in the SOD1 gene may impair antioxidant enzyme activity thereby leading to accumulation of toxic superoxide anions [10].

SOD1 gene has five exons and the +35A/C polymorphism (rs2234694) is adjacent to the splicing point (exon3/intron3), being related to macrovascular complications in diabetes and having no association with microvascular complications [4]. While data regarding the SOD1 (+35A/C) gene polymorphism is available for other populations, including North Indians, the Bangladeshi, the Finns, the Romanians, the New Zealanders, and the Czechs [4, 11–15], it is lacking among South Indian population. Therefore, the present study has been conducted to determine the prevalence of +35A/C polymorphism in intron3/exon3 boundary of the SOD1 gene among South Indian population. In addition, the genetic association between polymorphism in the (+35A/C) SOD1 gene and type 2 diabetes mellitus has also been studied.

## 2. Materials and Methods

### 2.1. Study Design

The study population consisted of seventy-five controls with no history of cardiovascular disease, diabetes, hypertension, cancer, or any infectious diseases and one hundred patients with type 2 diabetes mellitus, belonging to South Indian population. The patients included were characterized based on the alteration in blood sugar level (>126 mg/dL). The samples from the subjects were collected into EDTA coated tubes and the informed consent was obtained. Clinical data including information on duration of diabetes, presence of any complication, history of other disorders, age, gender, lipid profile, blood sugar level, and systolic and diastolic blood pressure were collected using a questionnaire. Ethical clearance was obtained for this study.

#### 2.1.1. DNA Extraction

Genomic DNA was extracted from the frozen blood by phenol-chloroform method [16]. For DNA extraction, 500 *μ*L of the blood was used and the isolated DNA dissolved in TE was stored at −20°C. The quality of the DNA was checked in 0.7 percent agarose (Hi-Media, Mumbai) gel electrophoresis and quantified using UV spectrophotometry (Hitachi, Japan).

#### 2.1.2. PCR Analysis of SOD1 (+35A/C) Gene [17]

PCR analysis was carried out using a thermal cycler (Eppendorf Mastercycler, Germany). Approximately 120 ng of genomic DNA was incubated in a total reaction mixture of 20 *μ*L containing both the forward primer 5′ CTATCCAGAAAACACGGTGGGCC 3′ and the reverse primer 5′ TCTATATTCAATCAAATGCTACAAAACC 3′ (~10 picomoles) (GenScript Corp., USA), 200 *μ*M deoxynucleotide triphosphate, 10x PCR buffer* p*H-8.3 containing MgCl_2_ 15 mM, and 5 units of* Taq* DNA polymerase (New England Biolabs, Beverly). DNA was initially denatured at 95°C for 8 min prior to amplification. The PCR amplification conditions were as follows: 30 cycles consisting of 1 min denaturation at 94°C, 50 sec annealing at 64°C, and 1 min extension at 72°C. The final extension included 7 mins at 72°C. The PCR product (278 bp) was confirmed by 2% agarose (Hi-Media, Mumbai) gel electrophoresis. The amplified product was used for further restriction fragment analysis.

#### 2.1.3. Restriction Enzyme Analysis [17]

Restriction digestion was performed in a total volume of 20 *μ*L consisting of 10 *μ*L amplicon, 4 *μ*L NE buffer (50 mM potassium acetate, 20 mM Tris-acetate, 10 mM magnesium acetate, and 1 mM dithiothreitol* p*H-7.9 at 25°C), and 10 U of* HhaI* enzyme (Fermentas Life Sciences, Germany). Samples were incubated for 6-7 hrs at 37°C and the digested PCR products were resolved in 2 percent agarose gel electrophoresis stained with ethidium bromide and separated bands were observed using gel documentation system. PCR-RFLP analysis revealed the existence of the three genotypes (AA, AC, and CC) of +35A/C SOD1 gene polymorphism.

#### 2.1.4. Sequencing of the PCR Amplified Product

The PCR product obtained was subjected to DNA sequencing which was carried out by Sanger's sequencing method (Synergy Scientific Services, Chennai) [18]. The DNA sequencing was done to check and confirm whether the amplified product was SOD1 gene sequence. The obtained sequence was then subjected to BLASTN analysis to study the homology sequence of the amplified product.

## 3. Results and Discussion

**Figure 1:**
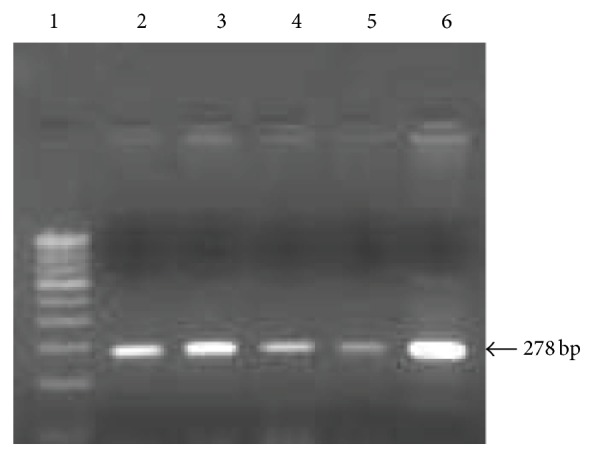
PCR analysis of SOD1 gene. Lane 1: 100 bp DNA ladder. Lanes 2–6: 278 bp PCR amplicon.

**Figure 2:**
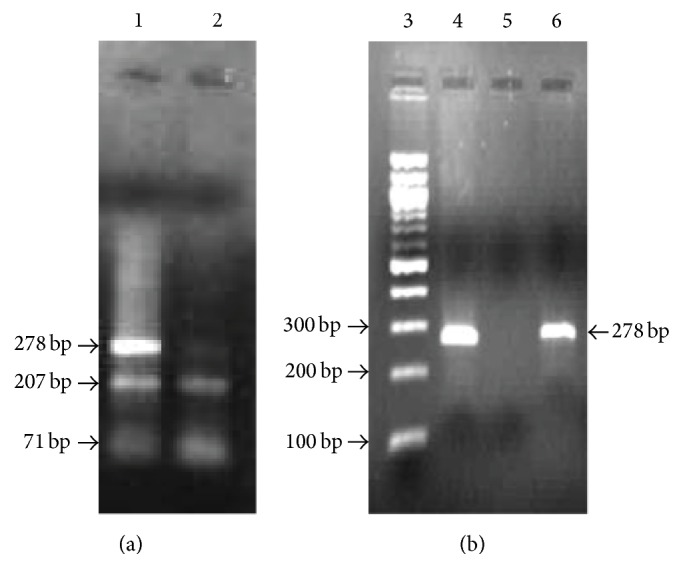
Restriction fragment length analysis of SOD1 gene +35A/C (exon3/intron3) polymorphism. Agarose gels (a) and (b) showing heterozygous (AC) genotype (Lane 1), homozygous mutant “CC” genotype (Lane 2), DNA marker-100 bp ladder (Lane 3), and homozygous wild-type “AA” genotype (Lanes 4 and 6).

The clinical data including the risk factors of study subjects are shown in Table 1. A 278 bp fragment that resides in the SOD1 gene was amplified (Figure 1) and sequenced. When BLASTN analysis was performed with the DNA sequence of PCR product (278 bp), 100 percent homology was found between the SOD1 gene and the submitted DNA sequence. After digesting the PCR amplicons with* HhaI*, the following fragment sizing patterns were observed by the gel electrophoresis: the SOD1 +35A/A genotype resulted in no cleavage of the amplified 278 bp fragments and the A/C genotype resulted in 3 fragments of 278, 207, and 71 bp. C/C genotype resulted in complete cleavage of the 278 bp fragment into 207 and 71 bp (Figure 2).

Genotype and allelic frequencies for the +35A/C SNP of the SOD1 gene in diabetic patients and nondiabetic healthy individuals were calculated. The results indicated that the occurrence of homozygous mutant (CC) and heterozygous mutant (AC) was completely absent in type 2 diabetes mellitus patients whereas such condition was observed in 4 (5%) and 6 (8%) of the controls (Table 2). As the two genotypes were completely absent in type 2 diabetes mellitus patients, Hardy-Weinberg equilibrium was not tested. The A allele frequency was found to be 1.0 for diabetic patients and 0.91 for controls. The C allele frequency was completely absent in patients when compared to controls (0.09), which indicates that the Cu/Zn SOD gene polymorphism may not be associated with the susceptibility to type 2 diabetes mellitus among South Indian population.

Similar result was observed in a study conducted among Finnish population that there is no difference in the genotype distributions and allele frequencies of the CuZn-SOD gene polymorphism between type 2 diabetes mellitus patients and controls [13]. Another study has also reported that the (CC) genotype and C allele were completely absent among North Indian population and that there is no association between diabetes and SOD1 +35A/C gene polymorphism [11]. However, contradictory results indicating that there is an association between diabetes and SOD1 +35A/C gene polymorphism were obtained in a study conducted among Bangladeshi population [12]. Similarly, Panduru et al. provided evidences that there is an association of SOD1 +35A/C gene polymorphism with diabetic nephropathy in Romanian population [14]. When the prevalence of the SOD1 +35A/C gene polymorphism among South Indian Tamil population was determined and the association of the mutant allele with type 2 diabetes mellitus was evaluated, no association was found between SOD1 +35A/C gene polymorphism and type 2 diabetes mellitus.

As allele frequencies are population specific, there is variation in allelic frequencies in different population (Table 3). The highest mutant allele frequency of SOD1 +35A/C gene polymorphism was observed among Czechs (0.4) and the lowest (0.018) was observed among South Indian population.

Both genetic and biochemical characterization of SOD1 [19] demonstrates that SOD1 gains importance in the development of diseases such as heart failure [20], cancer [21], diabetes [4], Down's syndrome [22], and amyotrophic lateral sclerosis [23]. It has been reported that the SOD's involvement in the pathogenesis of diseases is due to altered SOD activity and ROS concentration [24]. It was also found that the SOD1 gene polymorphism influences SOD activity [25]. Decreased activity of the antioxidant enzymes and depletion of total antioxidant capacity may increase the susceptibility of diabetic patients to oxidative injury [26]. Similarly, studies conducted by Sayed et al. and Fujita et al. have reported that the enzymatic activity of SOD was significantly decreased in diabetic patients with retinopathy [27] and nephropathy [28]. Clinical studies conducted in different populations have shown a decrease in SOD activity in type 2 diabetic patients when compared to controls [29, 30].

## 4. Conclusions

As the mutant genotype and allele frequency were completely absent in type 2 diabetic patients among South Indian population, it was concluded that the CuZn-SOD (+35A/C) gene polymorphism may not be associated with the susceptibility to type 2 diabetes mellitus. However, this lack of association might be due to smaller sample size and ethnic variation. Further study is needed to investigate the other SNPs (Mn-SOD and EC-SOD) in SOD gene to elicit the potential role of antioxidant defense and susceptibility in type 2 diabetes mellitus.

## Figures and Tables

**Table 1:** Clinical data of the study subjects.

Parameters	Type 2 diabetic patients (*n* = 100)	Controls (*n* = 75)
Age (mean ± SD)	49.67 ± 10.24	35.94 ± 11.89
Sex (M/F)	50/50	32/43
Hypercholesterolemia	47	—
Family history	45	—
Hypertension	18	—
Other complications	15	—

**Table 2:** SODI gene polymorphism: genotype and allele frequency in patients and controls.

Genotype/allele	Patients (*n* = 100)	Controls (*n* = 75)
Genotypes (+35A/C)		
AA	100	65
AC	—	6
CC	—	4
Alleles		
A	1.0	0.91
C	—	0.09

**Table 3:** Prevalence of the SOD1+35A/C gene polymorphism in different populations.

Country	Ethnic population	Number of subjects	Disease risk	Mutant allele frequency	References
India	South Indian	P-100 C-75	T2DM	P-0 C-0.09	(Present study)
India	North Indian	P-207 C-210	T2DM	P-0 C-0	[11]
Bangladesh	Bangladeshi	P-109 C-144	T2DM	P-0.018 C-0.014	[12]
Finland	Finns	P-239 C-245	T2DM	P-0.09 C-0.10	[13]
Romania	Romanian	P-106 C-132	T1DM	P-0.06 C-0.01	[14]
New Zealand	European	P-230 C-210	COPD	P-0.05 C-0.06	[15]
Czech Republic	Czechs	P-306 C-140	T2DM	P-0.42 C-0.48	[4]

P: patients; C: controls.
